# Guiding Similarity Search in Chemical Fragment Spaces with Weighted Fingerprints

**DOI:** 10.1021/acs.jcim.5c02952

**Published:** 2026-02-10

**Authors:** Justin Lübbers, Malte Schokolowski, Uta Lessel, Alexander Weber, Matthias Rarey

**Affiliations:** † 14915 University of Hamburg, ZBH-Center for Bioinformatics, Albert-Einstein-Ring 8-10, 22761 Hamburg, Germany; ‡ 60325 Boehringer Ingelheim Pharma GmbH & Co. KG, Global Medicinal Chemistry, Birkendorfer Straße 65, 88397 Biberach an der Riß, Germany

## Abstract

The introduction of chemical fragment spaces as a way to model large chemical spaces led to readily available compound libraries several orders of magnitude larger than seen before. The possibility of efficient similarity search based on molecular fingerprint comparison in such chemical fragment spaces was introduced by the SpaceLight algorithm for the first time. In this work, we introduce weighted SpaceLight, an enhancement that allows the algorithm to focus the search on important areas of a query molecule, increasing the local similarity while increasing variability in other areas, ultimately providing more structural control over the results. Due to the size of chemical fragment spaces, such customization methodologies become crucial to avoid millions of hits which have to be postfiltered. We demonstrate how weighted SpaceLight produces more molecules that preserve selected substructures during similarity search and how it can be adapted for different search scenarios. Combining global fingerprint similarity with a focus on specific substructures bridges the gap between existing search methods like SpaceLight and SpaceMACS and offers a new level of control for chemical space exploration in drug discovery.

## Graphical abstract

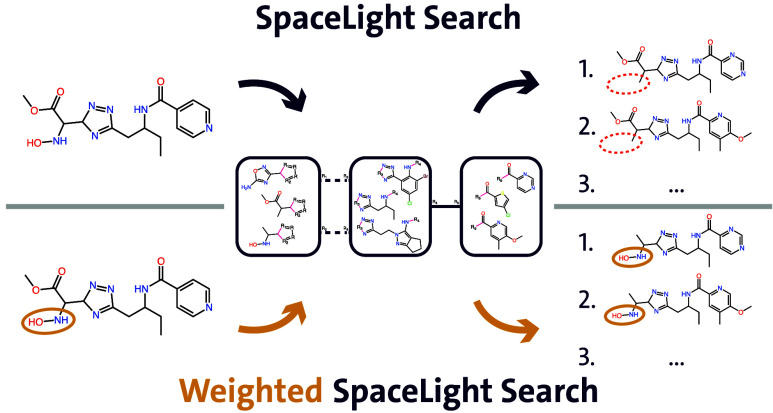

## Introduction

Virtual compound collections based on combinatorial chemistry have become an important resource in drug discovery. Make-on-demand catalogs like Enamine’s REAL Space^1^ (83 billion molecules), Ambinter’s AMBrosia^2^ (126 billion molecules) and Synple Chem’s recently published Synple Space^3^ (1 trillion molecules) offer digital and synthetic access to orders of magnitude more molecules than any traditional compound database. The on-demand synthesis and delivery accelerate workflows across many areas in the drug discovery workflow. The same technology is also used by major pharmaceutical companies to create their own proprietary chemical fragment spaces based on in-house synthesis knowledge, such as Boehringer Ingelheim’s BICLAIM,^4^ or Merck’s MASSIV,^5,6^ as well as for public domain compound collections such as the recently published SAVI Space.^7^

However, working with these vast collections is not trivial. Due to their size, they are practically not enumerable, making classic search and filter algorithms unusable. Hence, special algorithms are needed that leverage the combinatorial nature of these virtual libraries and do not rely on enumeration. The first algorithm of this kind was FTrees-FS.^8^ Recent examples of such algorithms are SpaceLight,^9^ SpaceMACS,^10^ and SpaceProp.^11,12^ SpaceLight is a tool for molecular fingerprint-based similarity search. With SpaceLight, it is possible to search trillion-sized chemical fragment spaces by fingerprint similarity in seconds on standard desktop hardware. Given a query molecule, the algorithm produces the most similar compounds that can be built from the building blocks and reactions that the fragment space encodes.

When using similarity search methods like SpaceLight to find molecules based on a query, there is often some domain knowledge about the query molecule that identifies specific areas of the molecule as more important than others. Examples are functional groups that form specific interactions or substructures that ensure a specific three-dimensional shape of the molecule, so it is able to fit into a protein binding site. In these cases, when searching similar molecules, often only those result molecules are relevant that preserve these key areas. Meanwhile, changes in other regions of the molecule may be desired. With SpaceLight, it is not possible to focus on specific substructures during the similarity search. FTrees-FS allows such focusing. However, it represents a fuzzy similarity concept for scaffold hopping, which is not substructure-precise. The alternative, SpaceMACS, offers common substructure and pattern search, but does not include overall similarity of the molecules. To bridge this gap, we have developed weighted SpaceLight, an extension to the SpaceLight algorithm that enables us to focus the similarity search on specific parts of the query molecules in a highly controlled manner.

## Methods

### Topological Fragment Spaces

Chemical fragment spaces encode molecules as building blocks with connection rules. There are several implementations of this concept. In this work, we use the topological fragment space representation introduced by Bellmann et al.^9^ A topological fragment space consists of a number of topology graphs. Each topology graph consists of a number of topology nodes and topology edges. A topology node contains a number of fragments. Each fragment contains one or more linker placeholder atoms with unique identifiers that represent atoms from connecting fragments. In addition to the named linker placeholder atoms, some fragments also contain unnamed ring placeholder atoms. Together with the named linker atoms, they are used to ensure that any rings that form when connecting multiple fragments are represented with their exact size in the fragments and their fingerprints. The topology edges define connections between topology nodes and, together with the unique identifiers of the linker placeholder atoms, determine how fragments can be combined to form complete molecules. Upon connection of the fragments, all placeholder atoms are replaced by real atoms of the connecting fragments as specified by the topology edges. Each topology edge also defines the bond type of the connection. Using this definition, a product molecule is built from a topology graph by choosing one fragment from each topology node and connecting them according to the information from the topology edges. An illustration is provided in Figure 1. The number of product molecules a topology graph encodes is equal to the product of the number of fragments in each topology node. Due to the combinatorial explosion, the number of products is typically several orders of magnitude larger than the number of building blocks.

**Figure 1:**
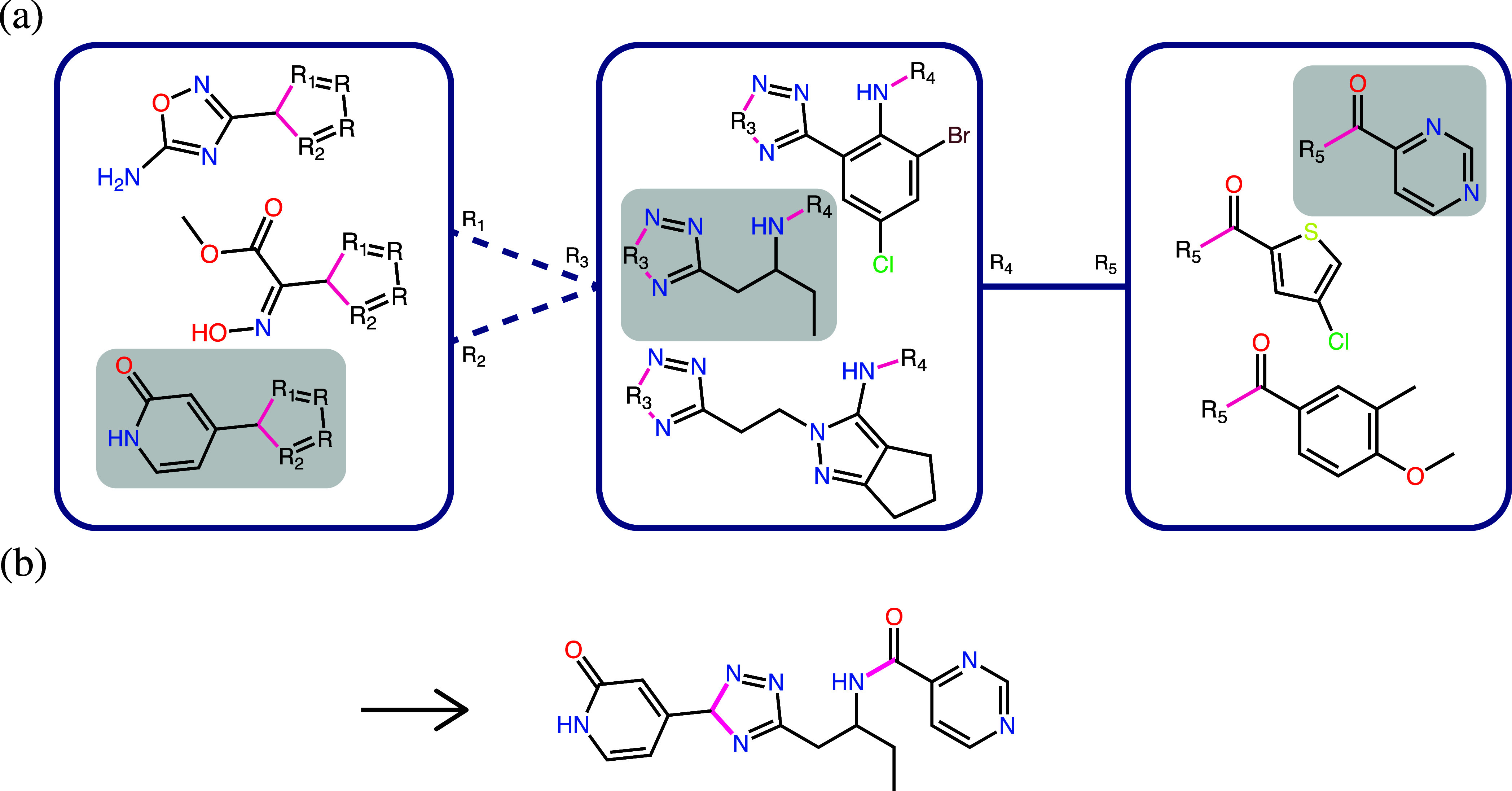
(a) Example of a topology graph. Three topology nodes, represented as blue boxes, are connected by topology edges, represented as lines. Depicted inside the nodes are the fragments containing linker placeholder atoms *R*_1–5_ and generic ring placeholder atoms *R*. The bonds that form a connection between fragments are highlighted in pink. The solid topology edge denotes a single bond, while the two dashed edges indicate an aromatic ring formation. Written on the edges are the linker identifiers that specify between which atoms the bond is formed and how the placeholder atoms are replaced. (b) An example product molecule which is formed by choosing the three highlighted fragments from the topology nodes and connecting them according to the topology edges.

### Molecular Similarity

There is a multitude of methods to describe and calculate molecular similarity.^13,14^ Within the context of this work, we use the Tanimoto coefficient,^15^ also known as Jaccard index, to compute similarity between molecular fingerprint vectors. A molecular fingerprint is a vector of bits. Each bit notes the presence or nonpresence of a molecular feature following a hashing strategy. Often, these features are derived from molecular substructures, as is the case for the ECFP^16^ and CSFP^17^ fingerprints, which are used by SpaceLight.^9^ The Tanimoto coefficient originally computes the similarity of two sets. The Tanimoto coefficient between two sets of features *A* and *B* is defined as the intersection of the two sets divided by their union

1 $$T\left(A,B\right)=\frac{\left|A\cap B\right|}{\left|A\cup B\right|}$$

The Tanimoto coefficient ranges from 0 to 1, where 0 represents no common features and 1 represents identity. For two bit vectors *x*, *y*, the formula can be defined as follows

2 $$T\left(x,y\right)=\frac{\sum_{i}\min\left(x_{i},y_{i}\right)}{\sum_{i}\max\left(x_{i},y_{i}\right)}$$

Note that this definition is a more general version of the Tanimoto coefficient, which is known as Ruzicka similarity.^18^ It is not restricted to bit vectors but is instead defined for two sequences of non-negative real numbers. It is also known as weighted Tanimoto coefficient or weighted Jaccard index.

### SpaceLight Algorithm

The SpaceLight algorithm^9^ is a similarity search algorithm for topological fragment spaces based on fingerprint similarity. Given a topological fragment space, a query molecule, and a desired number of compounds *N*, it produces the *N* most similar fragment combinations from the fragment space according to fragment combination similarity, supporting CSFP and ECFP fingerprints with various resolutions. To avoid the impractical enumeration of a fragment space, it operates mainly on the building blocks instead of full molecules. This enables SpaceLight to search large combinatorial libraries like Enamine’s REAL Space^1^ (83 billion molecules) in seconds, using typical values of N between 100 and 1,000,000. The algorithm consists of four steps:

1. *Partitioning Step:* First, the query compound is partitioned into connected substructures (partition classes). Since there are many possible partitions, only those are enumerated that resemble the topology of at least one topology graph of the given fragment space.
2. *Matching Step:* For each partition, the partition classes are matched onto the nodes of compatible topology graphs. A topology graph is compatible with a partition if the number of partition classes is equal to the number of nodes and the topology of the graph is equal to that of the partition, including bond types. Additionally, every partition class has to have the same connectivity as its corresponding topology node and must be similar in size to at least one fragment of the node. Here, similar in size is defined as a difference in heavy atom counts of at most 5 atoms. Every matching pair of partition and compatible topology graph that fulfills the criteria proceeds to the next step.
3. *Comparison Step:* For each found pair of partition and topology graph, the fragments of the topology nodes are compared to their corresponding partition class. This is done by calculating the Tanimoto coefficient^15^ for the molecular fingerprints of the fragments and the query substructure. The resulting partial similarity score is used to rank the fragments of the node by their similarity to the partition class. The result of this step is, therefore, a ranked list of fragments for each topology node for each matching of query partition and topology graph.
4. *Combination Step:* In the final step, the *N* best-scoring fragment combinations for all matchings from step two are assembled. To compare different combinations of fragments from different partition matchings, a combined score is calculated for every fragment combination. For one combination of fragments, this score consists of the weighted sum of the calculated partial similarity scores of each fragment (step 3), where the weight of each score is equal to the ratio of the number of heavy atoms of the respective partition class of the respective query partition and the total number of heavy atoms in the query molecule. As a result, the partial similarity of a fragment has more impact if the partition class covers a larger part of the query molecule. The combined score is then used to rank the fragment combinations and produce the final list of *N* most similar compounds.

These steps can be performed in parallel for all topology graphs within a topological fragment space. In the end, all top fragment combinations from each topology graph are combined into one list, before the best *N* solutions are returned. It is worth noting that in the implementation of SpaceLight used as a foundation of this work, which was provided by BioSolveIT, one additional step is optionally performed that was not part of the original algorithm. Before combining the solutions from each topology graph into the final result list, the global fingerprint similarity score between the query molecule and each fragment combination is calculated to achieve higher compatibility with sequential searching. The global similarity score is then used to rank the combinations and provide the final list of results.

### Weighted SpaceLight

In this work, we propose an extension to the SpaceLight algorithm that enables highlighting of specific parts of a query molecule, increasing their importance during the similarity calculation. As a result, we can guide the algorithm to pay specific attention to these parts, enriching the results of the search with molecules that conserve the desired structures while increasing variability in other areas and keeping overall similarity high. To accomplish this, we introduce weighted fingerprint matching to the SpaceLight procedure.

First, we mark the atoms of the query molecule that are part of the desired substructure. In this work, we use SMARTS expressions to select the atoms, but other methods, such as direct, interactive selection of atoms or indirect selection using maximum common substructures with other molecules, could be used instead. These selections are carried down the SpaceLight calculation pipeline to step 3, the *Comparison Step*. In this step, we compare the query substructures to fragments in the topology nodes. During the calculation of the fingerprints for the query substructures, we collect every fingerprint feature that consists completely of marked atoms in a set *M*. Then we define a weighting function *w* that assigns every feature *i* in *M* a user-controllable weight *k*_*i*_

3 $$w\left(i\right)=\{\begin{array}{ll} k_{i}, & i\in M\\ 1, & i\notin M \end{array}$$

When comparing the query substructure fingerprints to the fragment fingerprints, we use this weighting function *w* to compute the weighted similarity with an adapted version of the weighted Tanimoto coefficient

4 $$T_{w}\left(x,y\right)=\frac{\sum_{i}w\left(i\right)\cdot \min\left(x_{i},y_{i}\right)}{\sum_{i}w\left(i\right)\cdot \max\left(x_{i},y_{i}\right)}$$

This definition is equal to the Ruzicka similarity if the values of all marked features in the fingerprint were set to their respective weight *k*_*i*_. This effectively multiplies the impact that a marked feature has on the Tanimoto coefficient by *k*_*i*_. Figure 2 illustrates this process, showing how a fragment that preserves a marked area increases in score while the score of a fragment that does not preserve the area decreases. As a result, the fragments with higher similarities in the marked areas are preferred.

**Figure 2:**
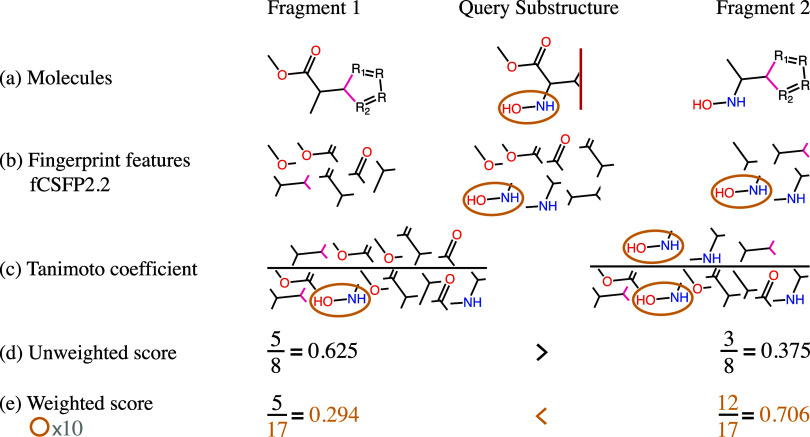
Example calculation of an unweighted and a weighted similarity score. (a) shows two fragments (Fragments 1 and 2) that are being compared to a query substructure in step 3 (comparison) of the SpaceLight algorithm. The yellow circle indicates the substructure marked for weighting. (b) shows the fingerprint features using the fCSFP2.2 descriptor (only features with two atoms) as an example. (c) demonstrates the separate calculation of the two Tanimoto similarities between fragment 1 and the query substructure (left) and between fragment 2 and the query substructure (right). The calculation is done by dividing the number of common features by the total number of features. (d) shows the calculation of the unweighted similarity score as done in the original SpaceLight algorithm, ignoring the highlighted substructure. (e) demonstrates how the similarity score changes when weighting the marked feature with a factor of 10. The numbers show that fragment 1 achieves a higher unweighted similarity score, but fragment 2, which contains the important substructure, achieves a higher weighted similarity score.

It is important to note that in this step, as defined by the original SpaceLight algorithm, the query is partitioned into disjunct substructures with no overlap. Therefore, during the comparison between a fragment and a query substructure, there is no knowledge about possible atoms beyond the linker atoms of the fragment or the query atoms beyond the borders of the query substructure. As a result, if the marked area for weighting is split across multiple query substructures, the comparison between a fragment and a query substructure can only consider a part of the marked area. Any features of the query that span across the partition borders can only be taken into account in the final, global comparison step of the algorithm. In this case, it is also important that only those marked features which are actually present in the query substructure are increased in weight. If there are marked areas outside of the given query substructure, their fingerprint features must not be increased in weight for the given comparison. If they were, a fragment could get penalized for containing such a marked feature because the increased weight of the feature would manifest in the denominator of eq 4 but not in the numerator, since the query substructure fingerprint does not contain the feature. For this reason, we calculate the marked features during the comparison step only for the given query substructure and not for the whole molecule. Another advantage of this approach is that the marked features are only enriched where they appear in the query and not in every fragment.

In the combination step, the fragment combinations are compared by their combination score. To reflect the importance of the marked regions, the impact of the partition classes that contain marked atoms must be increased in the weighted sum of the combination score. If not, fragment combinations with high similarity in marked regions can be surpassed if the partition class containing the marked region is small. An example of this problem is illustrated in Figure 3a–c. The example shows two query partitions (A and B), one where the partition class containing the marked area is small (A.2, 4/10 heavy atoms) and one where it is large (B.2, 8/10 heavy atoms). The calculation of the combined scores shows that although the top fragment combination originating from partition A has a higher partial similarity to the important partition class A.2 (1.0) than the other fragment combination to B.2 (0.409), its combination score is dominated by the fragment combination of partition B. This is because the important partition class A.2 is small, which decreases the impact of its fragments’ high partial similarity on the final score. To mitigate this problem, the importance of the marked areas has to be reflected in the weighted sum of the combined score.

**Figure 3:**
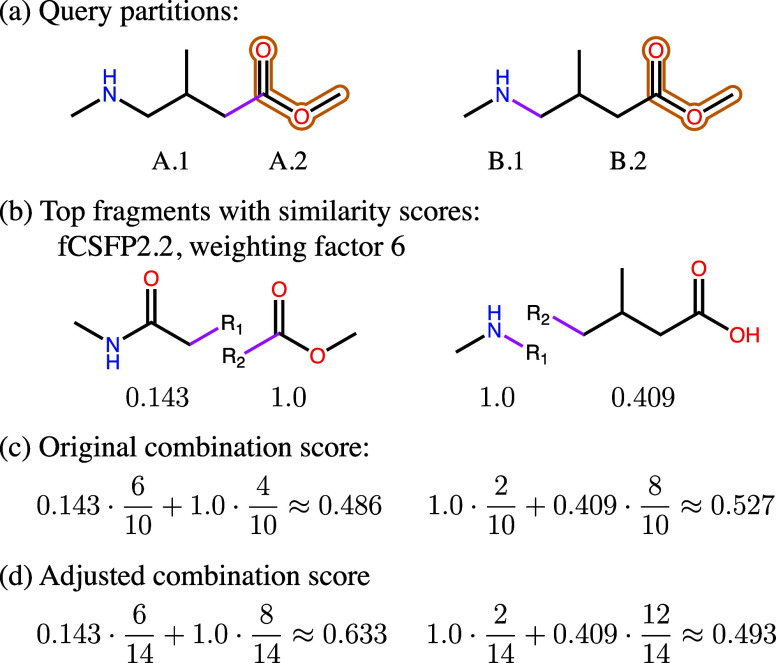
Example calculation of the original and the adjusted combination score. (a) shows two query partitions of the same query molecule with marked atoms (yellow). The bonds separating the partition classes in the partitions are marked in pink. (b) shows example top-scoring fragments from step 3 (comparison) with their weighted similarity scores using the fCSFP2.2 descriptor (only fingerprint features with two atoms) and a weighting factor of 6. (c) shows the calculation of the original combination score as the weighted sum of the similarity scores weighted by the share of heavy atoms of the respective query substructures. (d) shows the adjusted combination score, which counts each marked heavy atom in the query as 2 (weighting factor 6 divided by correction factor 3). The numbers show that the top-scoring fragment combination of query partition B achieves a higher original combination score, but the top-scoring fragment combination of query partition A achieves a higher adjusted combination score.

There are multiple ways to achieve this goal. In this work, we follow the approach of weighting by the number of heavy atoms that the original SpaceLight algorithm uses. When counting the number of heavy atoms of the partition classes, we count every marked atom not as one but as multiple atoms, depending on the weighting factor associated with the atom. However, we found that using the full weighting factor for this step focuses too strongly on the partition classes that contain these marked atoms and disregards all other parts of the query. Therefore, we divide the weighting factor of each atom by a correction factor before using it to count the atoms. Following some empirical testing, we chose a correction factor of 3 because of a good balance between preserving marked features and maintaining overall compound similarity. This way, the focus remains controllable and only depends on one parameter. Note that this is a very simple approach, and more sophisticated methods could be applied. However, we found that the performance of the algorithm did not improve for any of the more complex implementations that we used, which is why we kept the straightforward approach of a constant correction factor. Figure 3d continues the example combination score calculation with the adjusted combination score. The weighting factor for the depicted scenario is 6. Therefore, each marked atom is counted as 2 in the adjusted combination score. As demonstrated in the example calculation, increasing the impact of the partition classes that contain marked atoms leads to higher final scores for fragment combinations that have higher similarities in the marked regions.

After selecting the best fragment combinations based on the combination score, the weighted fingerprint similarity is also applied to the final ranking of the fragment combinations based on global fingerprints. In this step, the fragment combinations with the highest combination scores are assembled into full molecules and compared to the query. Here, we compute all fingerprint features of the marked areas and use them for the weighted fingerprint similarity calculation. The weighting in the final step is particularly important for larger marked areas. As described above, if the marked area spans multiple building blocks, the previous steps are only able to enrich parts of the marked area within these building blocks without ensuring that these parts connect correctly to form the larger structure of interest in the final molecule. In the final global comparison, however, previously unseen and potentially larger marked fingerprint features spanning multiple fragments can be taken into account. Their weight is also increased in this final comparison, which leads to higher scores for molecules that preserve the larger area of interest and lower scores for molecules that only contain unconnected parts of that area.

It is worth noting that this weighting process only increases the probability of finding molecules that contain a marked structure, but does not guarantee it. Fragments which contain the necessary parts of a marked structure in the correct configuration can be overshadowed by fragments that also contain the parts of the marked structure in a wrong configuration but have a higher overall similarity to the query substructure. If there are too many of these false positive fragments, the true positive fragments are not contained in the result list of the comparison step and, therefore, are not considered in the combination step. In practice, however, this is very rare and our experiments did not suggest a need for additional algorithmic steps for this specific scenario.

In theory, it is possible to select an arbitrary number of atoms for the weighting, potentially increasing the importance of large parts of a molecule during the search. In this case, however, it is important to consider the molecular fingerprint variant used for the search. To make the search of large topological fragment spaces feasible, all molecular fingerprints of the building blocks of the fragment spaces are precalculated and stored within a database. At the time of writing, the most recent versions of chemical fragment spaces provided by BioSolveIT^19^ contain ECFP-like fingerprints with a maximum diameter of 8 ^16^ and CSFP fingerprints with up to 5 heavy atoms per feature.^17^ That means, when using the fCSFP1.4 fingerprint and marking a substructure with 8 atoms within a query molecule for the weighted search, there is no single fingerprint feature that represents the full substructure. Instead, the weighting is only applied to smaller features with up to 4 atoms, that make up the larger structure. Another difficulty is the minimum feature size of the fingerprints. The smaller features with one or two atoms are significantly less specific than the larger features, so including them in the weighting potentially adds a lot of noise to the comparison. However, since larger substructures of interest can be split across multiple building blocks with only small portions contained within single fragments, it is nevertheless important to include the small features in the weighting process. To account for these factors, we additionally investigated two extensions of the weighting algorithm. The first is the use of different minimum feature sizes for the weighted fingerprint features. This reduces the number of less meaningful features in the weighting. The second extension is to multiply the weight for each fingerprint feature by the number of heavy atoms contained in it. This increases the relative importance of the larger substructures compared to the smaller ones.

## Results

### Validation

To evaluate whether the presented approach is capable of focusing on specific parts of molecules, we performed traditional and weighted SpaceLight searches with combinations of random query molecules and random substructures for weighting. The substructures were extracted as SMARTS patterns. Afterward, we analyzed whether the result molecules preserved the selected substructures by using the SMARTS patterns and compared the weighted to the unweighted results. The random molecules were taken from ChEMBL (Version 35)^20^ with the restriction of having at most one violation of Lipinski’s Rule of Five^21^ criteria and only one connected component. All searches were done in Enamine’s REAL Space^1^ (downloaded 10/2024, 70 billion molecules) as well as the SAVI Space^7^ (7 billion molecules) with 10,000 result molecules. We used weighting factors 5, 10, 15, and 20 for weighted searches. We conducted two basic experiments, one with the fCSFP1.4 descriptor, which is the default for the SpaceLight software, and one with the ECFP_4 descriptor. We performed three additional experiments to analyze the effect of using a higher minimum feature size for the weighting (2 and 3), as well as the weighting by size approach using the fCSFP1.4 descriptor.

The query molecules and SMARTS patterns were generated based on the following specifications. To account for different substructure sizes, we generated patterns that match 3 to 7 connected heavy atoms of their respective query molecules. We enforced that ring structures could only be included as a whole and that at least one atom of any substructure was not carbon. Additionally, we discarded combinations of molecules and SMARTS patterns based on the following two criteria. First, if neither the unweighted nor the weighted searches produced any molecules that matched the SMARTS pattern, it can be assumed that the target fragment space simply does not contain molecules that are similar to the query and contain the given substructure. These data points are not useful for evaluating the presented approach. Second, if all molecules found by the unweighted search already contain the structure, the weighted search cannot provide any benefit. These data points do not represent the intended use case and are therefore also not included in the evaluation.

We generated 500 pairs of molecules and SMARTS patterns for each of the five experiments, consisting of 100 pairs for each atom count from 3 to 7. Some example molecules as well as the complete query data sets are provided in the Supporting Information (Figure S1). The results of the experiments for the basic weighted search in the REAL Space are presented in Figure 4. On average, 50% of the result molecules produced by the unweighted search contain their respective substructure. When using the weighting approach 77% (5), 88% (10), 90% (15), and 91% (20) of molecules preserved the substructures. The results show that, on average and for each tested pattern size, the weighted approach clearly outperforms the standard search in terms of preserving chosen substructures. As anticipated, the performance for both approaches is influenced by the pattern size. In most of the experiments, the preservation score, meaning the share of result molecules that preserve the given substructure, decreased with increasing pattern size from 3 to 5 atoms. For the weighted approach, this score decreased further for 6 and 7 atom patterns.

**Figure 4:**
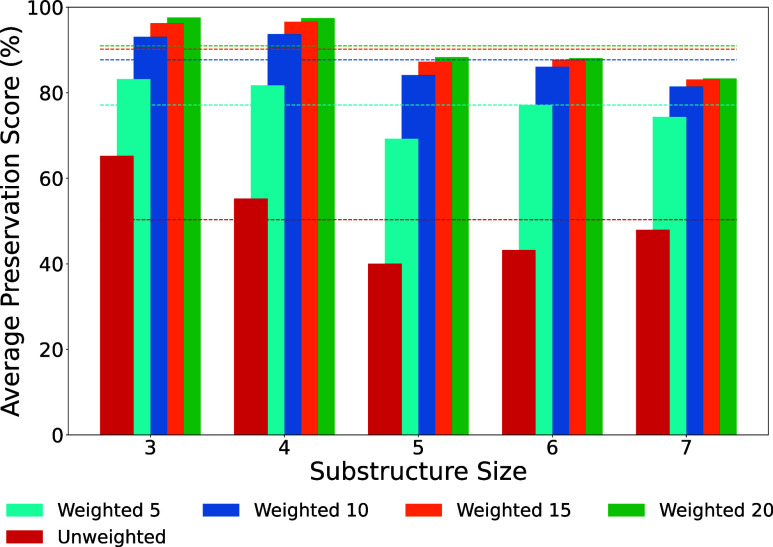
Statistical validation results for weighted SpaceLight using the fCSFP1.4 descriptor. The average preservation score (the share of result molecules that preserve the given substructure) is shown for substructure sizes 3 to 7. The average rate over all substructure sizes for the different searches is indicated as a dashed line.

The detailed results for the remaining experiments are provided in the Supporting Information (Figures S2 and S3). In summary, they generally yielded similar results. With a minimum feature size of 2, the search achieved a slightly higher preservation score for the large patterns with 7 atoms than the basic weighted search. However, increasing the minimum feature size to 3 did not produce better results. Instead, the preservation scores for the smaller patterns with 3 atoms dropped slightly. The weighting by feature size approach is on the same level as the minimum feature size 2 search, although similar preservation scores are reached with lower weighting factors. This is expected because for a weighting factor of 5, the actual multipliers used in the algorithm range from 5 to 20 when using the fCSFP1.4 descriptor, depending on the size of a given fingerprint feature. When using the ECFP_4 descriptor, the weighted approach also outperforms the unweighted search. However, the effect is smaller than for the fCSFP1.4 descriptor. The unweighted search reached an average preservation score of 49% in the REAL Space while the weighted search reached 77% with a weighting factor of 20.

All in all, the experiments show that weighted SpaceLight significantly improves the algorithm’s ability to preserve given substructures during search across both tested descriptors. The augmentations of the weighting did not make a significant difference in the statistical evaluation. However, using a minimum feature size of 2 and adapting the weighting factors to the size of the fingerprint features might provide some benefit for specific use cases with large areas of interest.

### Runtime

The presented weighted SpaceLight extension costs computational resources for calculating the fingerprint features of the marked atoms and for the lookup of feature weights during fingerprint comparison. These increased costs lead to a moderate increase in runtime, as Figure 5 shows. The experiments were executed on an Apple MacBook Pro with an M3 Pro processor using all 11 cores for parallel computing. We performed 100 searches in the REAL Space^1^ (downloaded 10/2024, 70 billion molecules) and the SAVI Space^7^ (7 billion molecules) and calculated the average runtime. The maximum memory consumption over all searches was approximately 5.1 GB for the REAL Space and approximately 2.7 for the SAVI Space. The weighted search had no significant impact on the memory consumption. It is important to note that the extension of the algorithm does not significantly change the scaling behavior of the original SpaceLight algorithm, which mainly depends on the number of fragments and not on the total number of product molecules encoded by the fragment spaces. Therefore, all currently available fragment spaces can be searched within seconds or minutes on standard consumer hardware.

**Figure 5:**
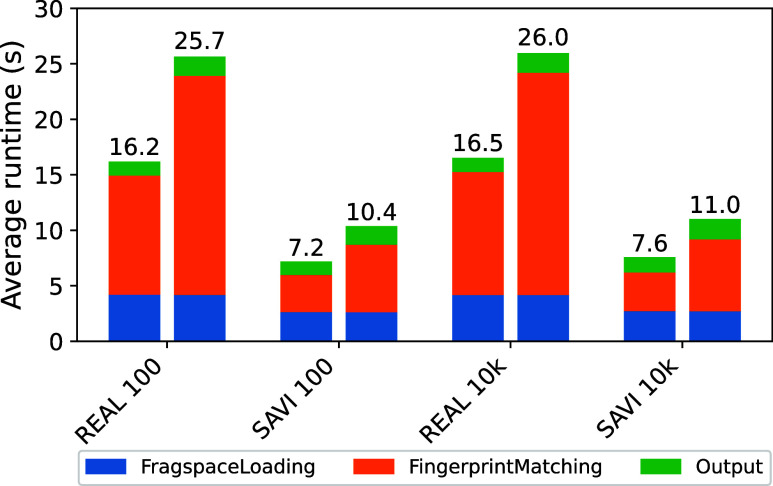
Runtime comparison for unweighted (left) and weighted (right) searches in REAL Space^1^ (downloaded 10/2024, 70 billion molecules) and SAVI Space^7^ (7 billion molecules) with 100 and 10,000 result molecules.

### Application Scenario

To show a possible application scenario, we performed an analog search with a glucosyltransferase (Gtf) inhibitor, which was also used by Schmidt et al.^10^ as an application scenario for SpaceMACS. The compound G43 was discovered by Zhang et al.^22^ as a lead compound targeting *Streptococcus mutans*, the main etiological agent of dental caries. After discovery, Nijampatnam et al.^23^ performed SAR studies on G43 in which they tested 90 analogs, the result of replacing two parts (left and right) with alternative structures as shown in Figure 6. Nijampatnam et al.^23^ based the structural modifications for the SAR studies on a docking model of G43 in the active site of Gtf. They used 10 different bicyclic structures for the left part and 9 phenyl rings with different substituents for the right part. The original experiments^22^ and the docking model suggested that the ortho-amide group is crucial for the activity of G43. However, Nijampatnam et al.^23^ found that some functional groups could further increase the potency of the compound.

**Figure 6:**
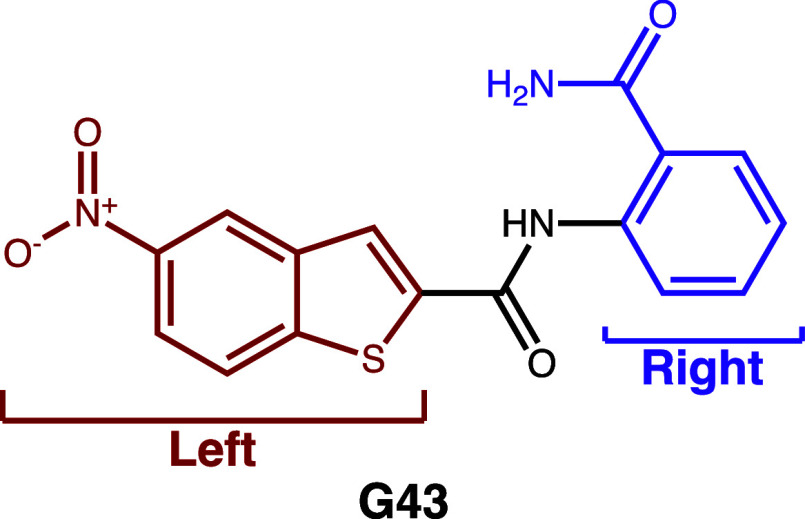
Compound G43 with two parts modified in SAR studies by Nijampatnam et al.^23^ (Adapted from Nijampatnam et al.^23^ Copyright 2020 American Chemical Society).

To demonstrate that weighted SpaceLight is capable of finding more relevant compounds in cases of specific structural requirements, we used G43 as a query to search for analogs. We defined the four SMARTS patterns *a*-*d* shown in Figure 7 to consider different use cases. All patterns capture the amide bond and the phenyl ring with a substituent in the ortho position. For the left side, patterns *a* and *c* only define one arbitrary atom in a ring connected to the amide bond. They are intended to explore any cyclic structure as a substituent for the left end of the amide bond. The other patterns *b* and *d* capture three arbitrary ring-atoms, specifying that the last atom has to be part of two rings. This way, the patterns ensure a polycyclic structure for the left part. In the right part, patterns *a* and *b* specify any heavy atom in the ortho position of the phenyl ring, which allows the exploration analogs with different ortho substituents. Patterns *c* and *d* capture the entire amide group, preserving the potentially crucial functional group entirely. The exact definition of the SMARTS patterns is as follows:

- *a*: [*;r]­C­(=O)­[N;H]­c1:[c;H]:[c;H]:[c;H]:[c;H]:c:1[!#1]
- *b*: [*;R2]∼[*;r]∼[*;r]­C­(=O)­[N;H]­c1:[c;H]:[c;H]:[c;H]:[c;H]:c:1[!#1]
- *c*: [*;r]­C­(=O)­[N;H]­c1:[c;H]:[c;H]:[c;H]:[c;H]:c:1C­(=O)­[N;H2]
- *d*: [*;R2]∼[*;r]∼[*;r]­C­(=O)­[N;H]­c1:[c;H]:[c;H]:[c;H]:[c;H]:c:1C­(=O)­[N;H2]

**Figure 7:**
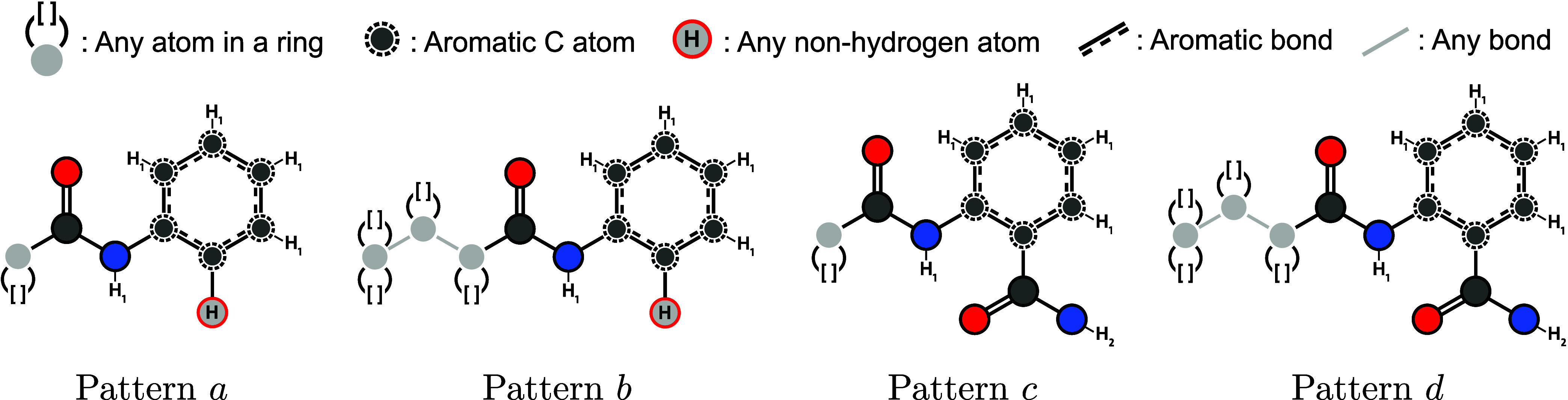
SMARTS patterns *a*-*d* used for the G43 analog search. Depictions were generated by the SMARTSviewer tool^24,25^ and adapted.

We performed five searches with G43 in the REAL Space^1^ (downloaded 10/2024, 70 billion molecules) and the SAVI Space^7^ (7 billion molecules), one unweighted search and one weighted search for each scenario. All searches were set to produce 10,000 results. The weighted searches were executed with a weighting factor of 20 and a minimum feature size of 2. Afterward, we used the four SMARTS patterns to determine how many matching compounds were produced by the searches. The results are displayed in Table 1. The Query column shows the parts marked for each weighted search. Note that in order to select only the highlighted atoms for the weighted search, the patterns *b* and *d* had to be slightly adapted. If used as described, the definition of the three atoms in the two-ring system would match all five atoms of the smaller ring of the query instead of only three atoms. To prevent this and only mark the areas shown in Table 1, the three ring atoms of pattern *b* and *d* were specified as carbon atoms for the input of the weighted search. For the evaluation, they were kept as arbitrary to include any 2-ring system in that position.

**Table 1:**
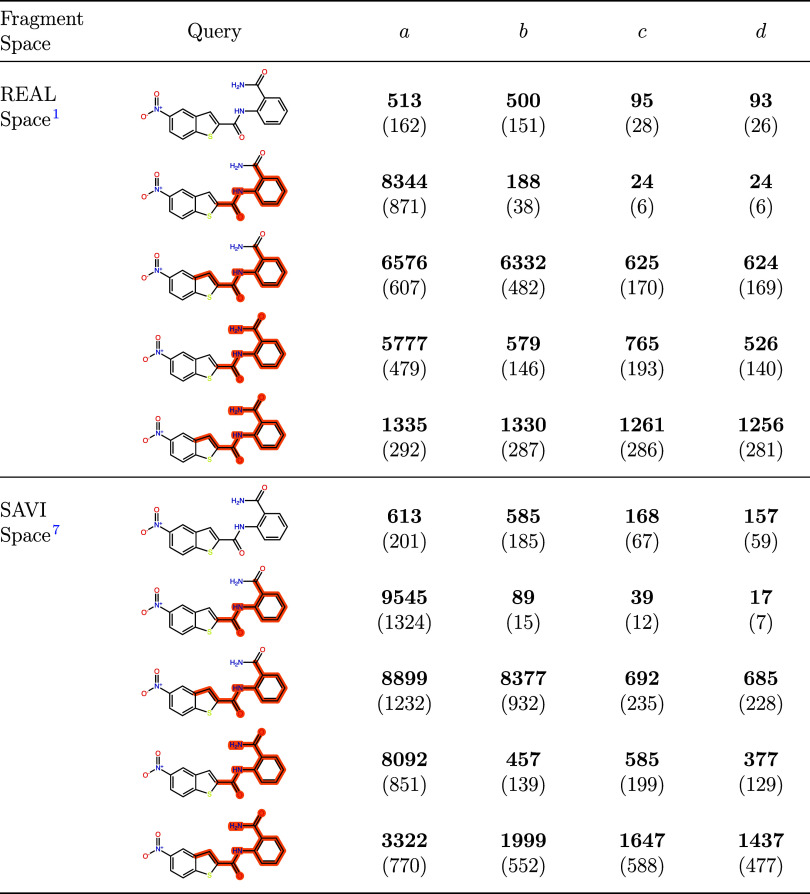
Evaluation of Unweighted and Weighted Search Results for G43 Analogs in the REAL Space^1^ (Downloaded 10/2024, 70 Billion Molecules) and the SAVI Space^7^ (7 Billion Molecules)^a^

a The query column shows the query for each search, consisting of compound G43 together with the selected atoms for the weighting (no highlight for the unweighted search). The columns a–d show the number of compounds in the 10,000 result molecules of the search that match the respective patterns and the number of distinct atom-based Bemis-Murcko scaffolds^26^ present in the matching molecules (in parentheses).

The first observation is that the default SpaceLight search, not aware of the importance of the patterns, only produces a reasonable, but low number of molecules that contain any of the patterns. In the results from the REAL Space, 5% of the 10,000 compounds contain pattern *a*, which is the least specific of the four patterns, and less than 1% contain pattern *d*, the most specific. In the SAVI Space, the numbers are in the same range. For the weighted search with pattern *a*, we see a significant increase of molecules that contain pattern *a* both in the REAL Space (83%) and in the SAVI Space (95%). We can also see the effect of the small marked part, since only very few molecules with bicyclic structures on the left or ortho-amide groups on the right or both were found, which were not included in pattern *a*. When searching with pattern *b*, which includes the bicyclic structure on the left, the number of molecules containing a bicyclic structure as well as a phenyl ring with an ortho substituent increased significantly (63% REAL Space, 83% SAVI Space). Although the ortho-amide group on the right is not included in the pattern, the number of molecules containing such a structure also increased. The weighted search with pattern *c* further increased this number in the REAL Space. However, it decreased in the results from the SAVI Space. Finally, searching with pattern *d* provided the highest number of molecules matching both the ortho-amide group on the right as well as the bicyclic structure on the left in both the REAL Space (12%) and the SAVI Space (14%). Additionally, it is worth noting that the differences in the number of relevant molecules are also reflected in their atom-based Bemis-Murcko scaffolds,^26^ which suggests that the found molecules are chemically diverse. Figure 8 shows the best-scoring molecules from the different searches that were not found in the 10,000 compounds from the unweighted SpaceLight search. More found scaffolds for each search are presented in the Supporting Information (Figures S4 and S5). To further assess the diversity of the found molecules, we calculated pairwise similarity distributions. The results show generally low similarity values, indicating chemical diversity and low redundancy among the compounds. The distributions can be found in the Supporting Information (Figures S6 and S7).

**Figure 8:**
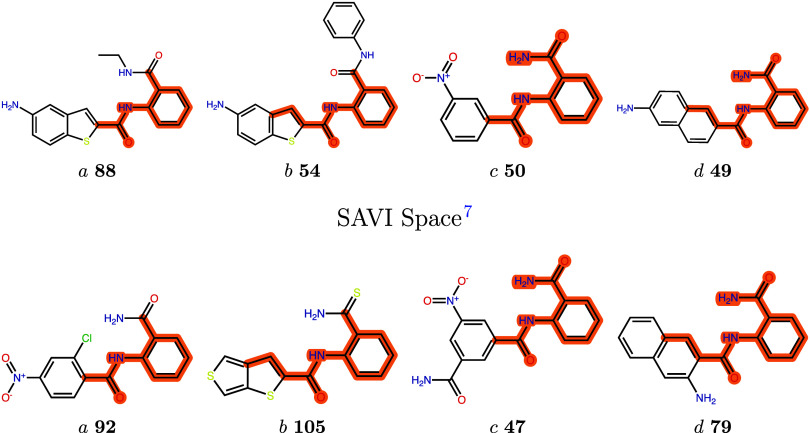
Best scoring compounds from weighted searches *a*-*d* in the REAL Space^1^ (downloaded 10/2024, 70 billion molecules) and the SAVI Space^7^ (7 billion molecules) that were not found in the 10,000 molecules from the unweighted search. The ranks of the molecules in the weighted search hit lists are given in bold numbers. The atoms matching the respective patterns *a*-*d* are highlighted.

## Conclusions

Fingerprint-based similarity search is a powerful tool for drug discovery workflows. The introduction of the SpaceLight algorithm enabled such similarity search in large chemical fragment spaces, increasing the possible search spaces for fingerprint similarity searches by orders of magnitude. In this work, we present an extension to the SpaceLight algorithm that uses weighted fingerprints to guide the focus of the similarity search toward the most important parts of the query molecules. This approach increases the control over the search and allows researchers to use their project-specific knowledge. In this frequent scenario, the algorithm avoids cascading a substructure search and a similarity search, which is usually highly inefficient, since the number of hits from a substructure search in a chemical space can be huge. The results demonstrate that our weighted SpaceLight implementation is capable of enriching substructures in the result molecules of a search. In addition, the application scenario showed that the method can be adapted to the specific demands of a project, enabling guided exploration of the chemical space around the query molecule. Potentially, the substructure recovery rate could be increased even further by performing repeated searches with different weighting patterns and pooling the results, which poses a promising direction for future research. All in all, the weighted SpaceLight approach is a powerful extension to a widely used algorithm that can benefit researchers in various stages of drug discovery workflows.

## Supplementary Material





## Data Availability

The weighted similarity search feature will be part of the SpaceLightN tool, available for Linux, MacOS, and Windows as part of the NAOMI ChemBio Suite at https://uhh.de/naomi for noncommercial fragment spaces such as the SAVI Space. It will be free for academic use and evaluation purposes. The SAVI Space can be accessed at https://www.fdr.uni-hamburg.de/record/15990.

## Notes

Molecules from the statistical validation experiment (Figure S1); additional results from the statistical validation experiment (Figures S2 and S3); example molecules from the G43 analog searches with different scaffolds (Figures S4 and S5); pairwise similarity distributions of the G43 analog search results (Figures S6 and S7) (PDF)
A content overview and detailed instructions on how to reproduce the G43 analog searches; (Statistical_Validation/Query_Data/)­All query molecules and corresponding SMARTS patterns from the statistical validation experiments; (Statistical_Validation/Example_Results/) All results from two example searches from the statistical validation, one from the REAL Space and one from the SAVI Space. Additionally, the molecule.smi file, the corresponding SMARTS pattern, an image showing the matching atoms, and a CSV file counting the number of pattern matches and unique Bemis-Murcko scaffolds are provided; (Application_Scenario/) All results from the G43 analog searches, as well as the script to count the number of pattern-matching molecules and their unique Bemis-Murcko scaffolds, the G43 SDFile, the SMARTS patterns *a*-*d* used for evaluation, and the CSV file containing the data for the application scenario evaluation table (ZIP)
