# Supplementary material for: Guiding Similarity Search in Chemical Fragment Spaces with Weighted Fingerprints
Source: J Chem Inf Model. 2026 Feb 10;66(4):2220–9. doi: 10.1021/acs.jcim.5c02952 (PMC12933719; doi:10.1021/acs.jcim.5c02952)
Supplement: Supplementary file 1 [file ci5c02952_si_001.pdf]

Supporting Information

Guiding Similarity Search in Chemical Fragment Spaces with Weighted Fingerprints

Justin Lübbers,† Malte Schokolowski,† Uta Lessel,‡ Alexander Weber,‡ and Matthias Rarey*,†

†*University of Hamburg, ZBH - Center for Bioinformatics, Albert-Einstein-Ring 8-10, 22761 Hamburg, Germany*

‡*Boehringer Ingelheim Pharma GmbH & Co. KG, Global Medicinal Chemistry, Birkendorfer Straße 65, 88397 Biberach an der Riß, Germany*

E-mail: matthias.rarey@uni-hamburg.de

This Supporting Information file contains the following items:

1. Example molecules from the statistical validation experiment (Figure S1).
2. Additional results from the statistical validation experiment (Figures S2 and S3).
3. Example molecules from the G43 analog searches with different scaffolds (Figures S4 and S5).
4. Pairwise similarity distributions of the G43 analog search results (Figures S6 and S7).

The Supporting_Information_Data.zip archive contains the following items:

1. (README.txt) A content overview and detailed instructions on how to reproduce the G43 analog searches.
2. (Statistical_Validation/Query_Data/)All query molecules and corresponding SMARTS patterns from the statistical validation experiments.
3. (Statistical_Validation/Example_Results/) All results from two example searches from the statistical validation, one from the REAL Space and one from the SAVI Space. Additionally, the molecule .smi file, the corresponding SMARTS pattern, an image showing the matching atoms, and a CSV file counting the number of pattern matches and unique Bemis-Murcko scaffolds are provided.
4. (Application_Scenario/) All results from the G43 analog searches, as well as the script to count the number of pattern-matching molecules and their unique Bemis-Murcko scaffolds, the G43 SDFile, the SMARTS patterns *a*-*d* used for evaluation, and the CSV file containing the data for the application scenario evaluation table.

Examples of validation molecules

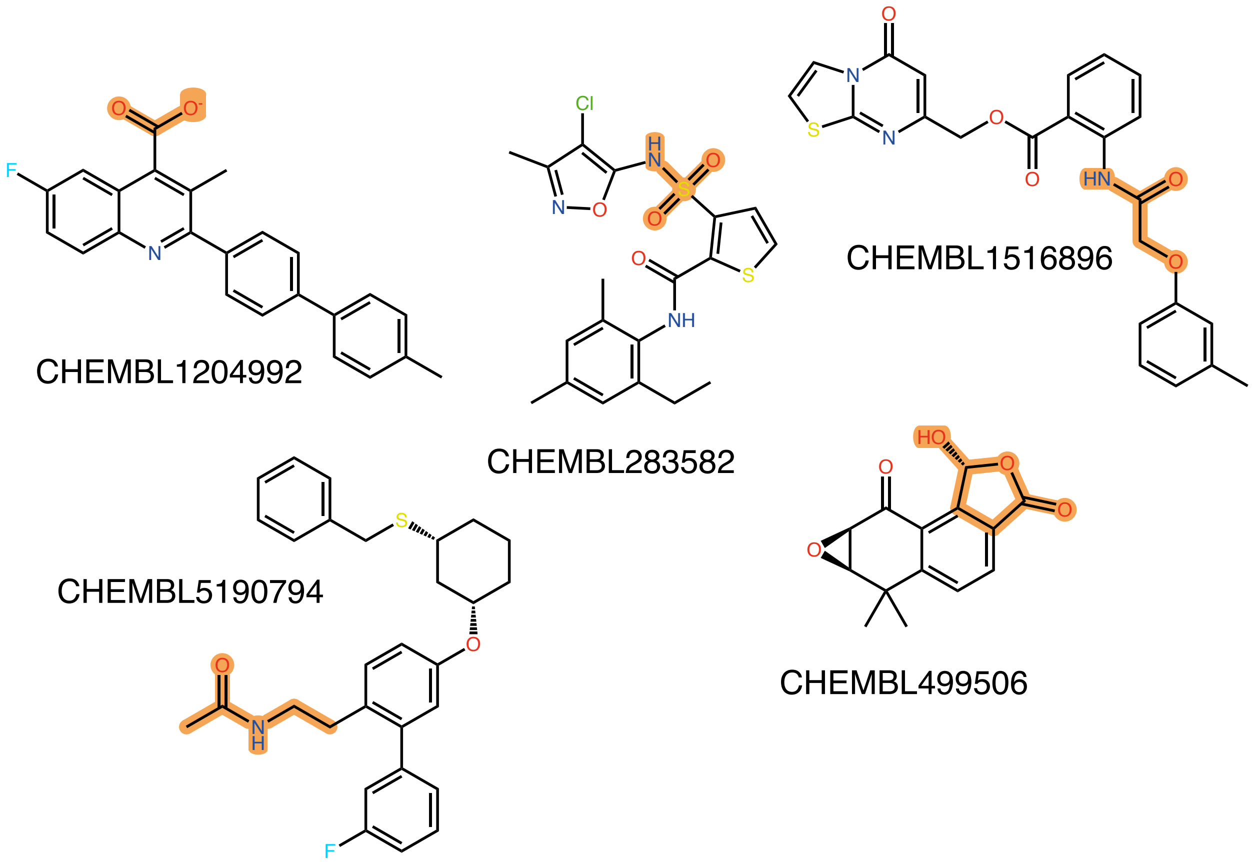

Figure S1: Example molecules from ChEMBL (Version 35)[1] used in validation experiments. The highlighted areas represent the substructures used for weighting and scoring.

Additional Validation Results

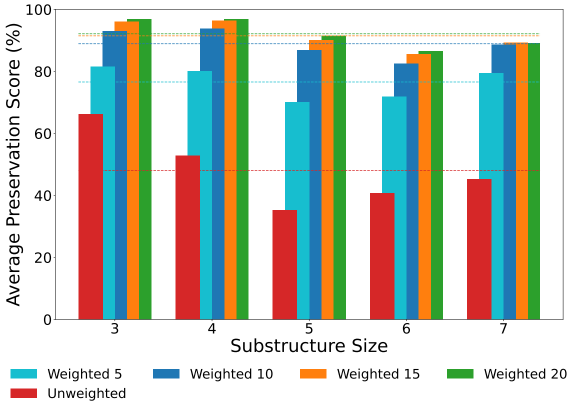

(a) Minimum feature size 2

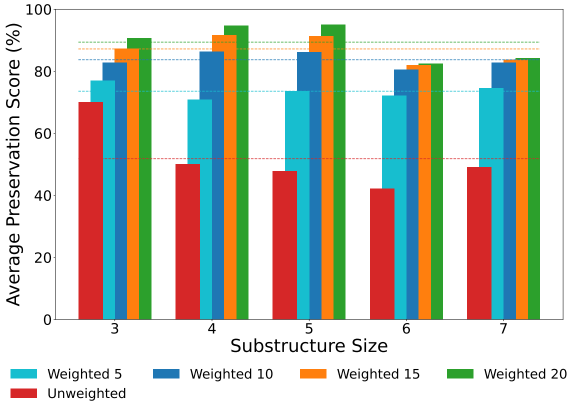

(b) Minimum feature size 3

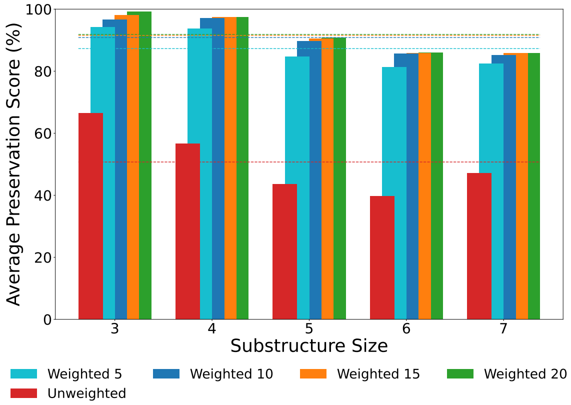

(c) Weighting by feature size

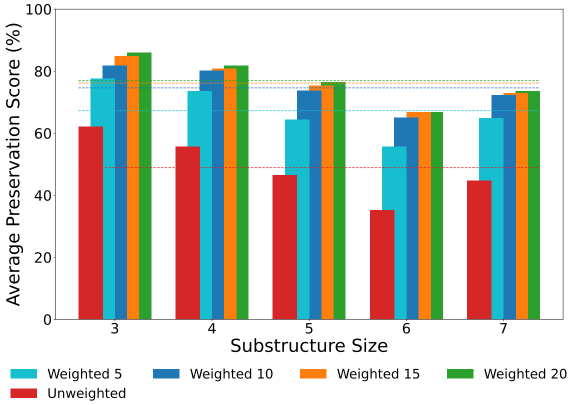

(d) ECFP_4

Figure S2: Additional statistical validation results in the REAL Space[2] (downloaded 10/2024, 70 billion molecules). The average preservation score (the share of result molecules that preserve the given substructure) is shown for substructure sizes 3 to 7. The average rate over all substructure sizes for the different searches is indicated as a dashed line. (a)-(b) show the results for the fCSFP1.4[3] descriptor with a minimum weighting feature size of 2 and 3, respectively. (c) shows the results for the weighting by feature size approach, also using the fCSFP1.4 descriptor. (d) shows the results for the validation experiment using the ECFP_4[4] descriptor.

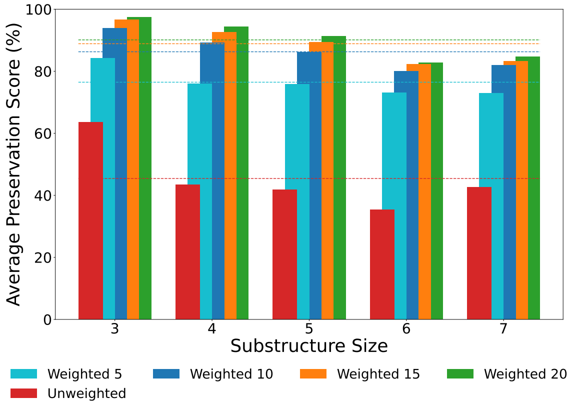

(a) Minimum feature size 1 (default)

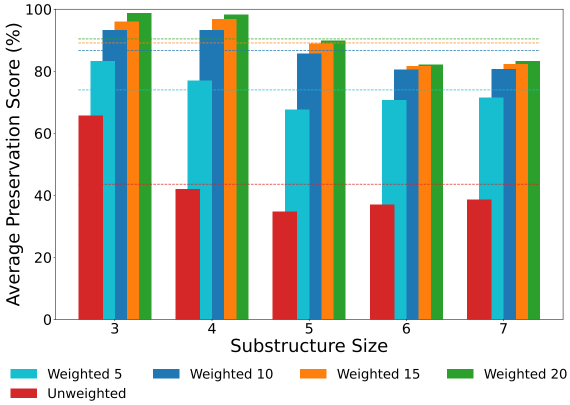

(b) Minimum feature size 2

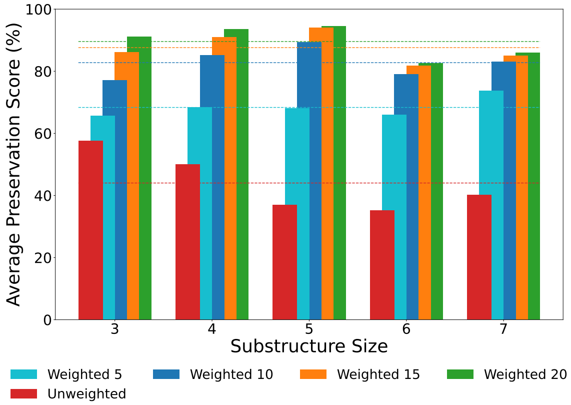

(c) Minimum feature size 3

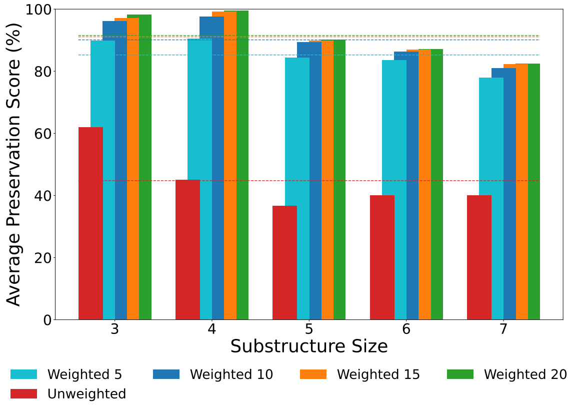

(d) Weighting by feature size

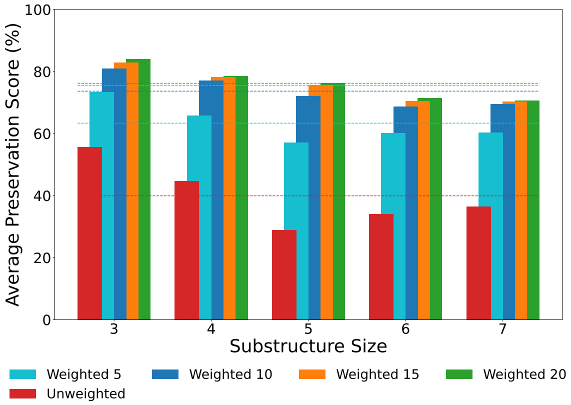

(e) ECFP_4

Figure S3: Additional statistical validation results in the SAVI Space[5]. The average preservation score (the share of result molecules that preserve the given substructure) is shown for substructure sizes 3 to 7. The average rate over all substructure sizes for the different searches is indicated as a dashed line. (a)-(c) show the results for the fCSFP1.4[3] descriptor with a minimum weighting feature size of 1, 2, and 3, respectively. (d) shows the results for the weighting by feature size approach, also using the fCSFP1.4 descriptor. (e) shows the results for the validation experiment using the ECFP_4[4] descriptor.

G43 Analog Search Scaffold Examples

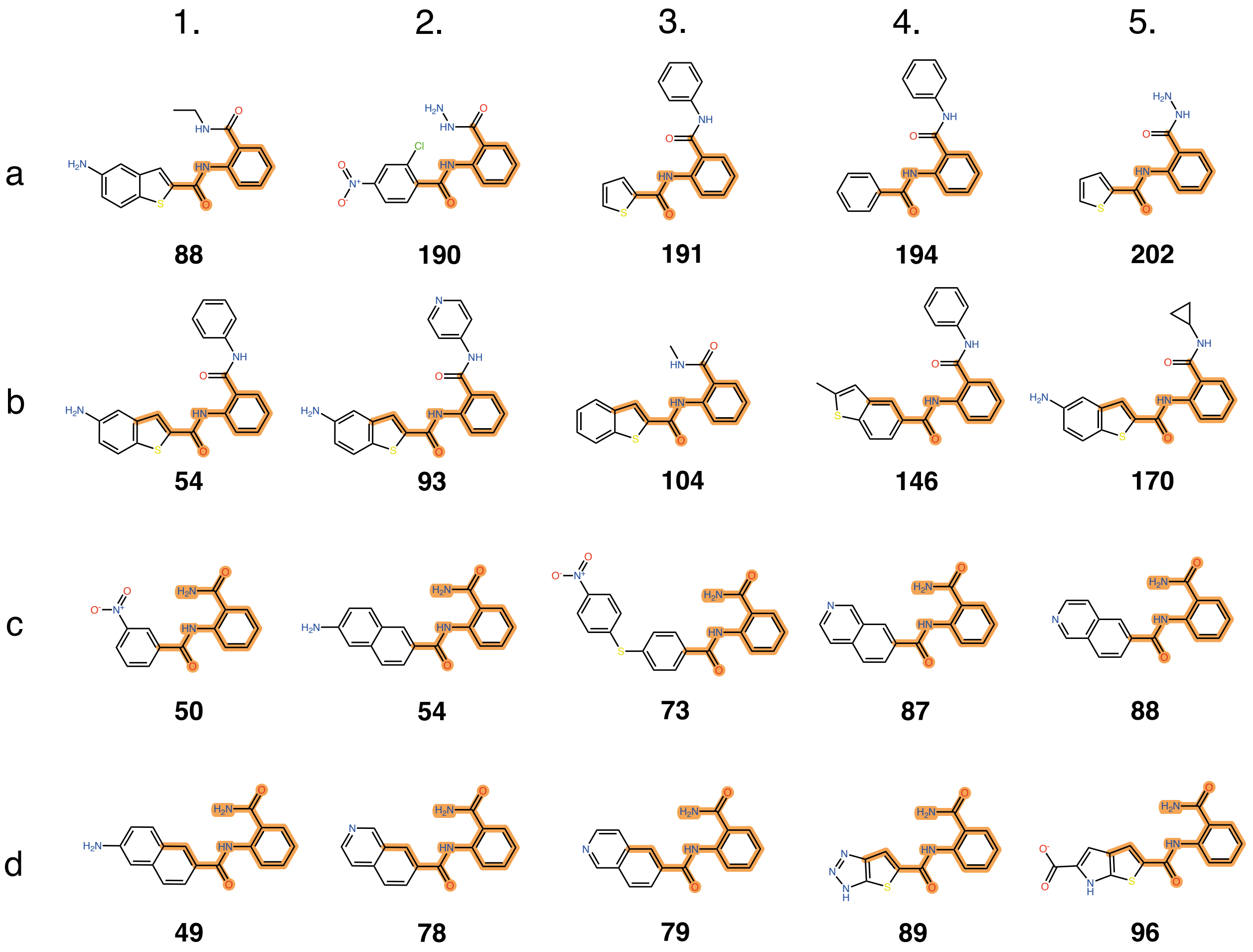

Figure S4: The top 5 scaffolds found by the weighted searches for G43 analogs using the weighting patterns *a*-*d* in Enamine's REAL Space[2] that were not included in the 10,000 results from the unweighted search. Each molecule represents the first found instance of the respective atom-based Bemis-Murcko scaffold[6]. The bold numbers represent the rank of the molecules in the result lists of the weighted searches.

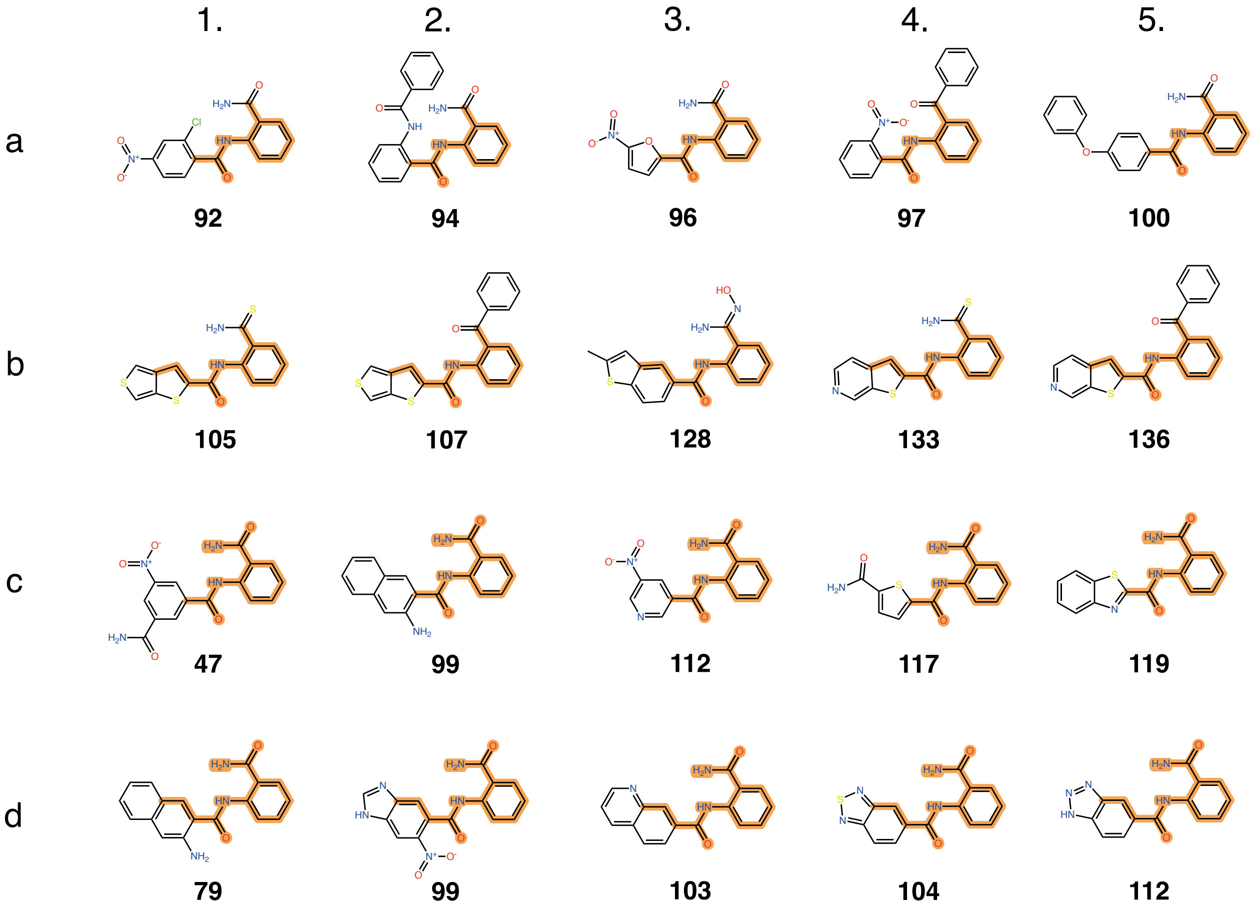

Figure S5: The top 5 scaffolds found by the weighted searches for G43 analogs using the weighting patterns *a*-*d* in the SAVI Space[5] that were not included in the 10,000 results from the unweighted search. Each molecule represents the first found instance of the respective atom-based Bemis-Murcko scaffold[6]. The bold numbers represent the rank of the molecules in the result lists of the weighted searches.

G43 Analog Search Results Pairwise Similarities

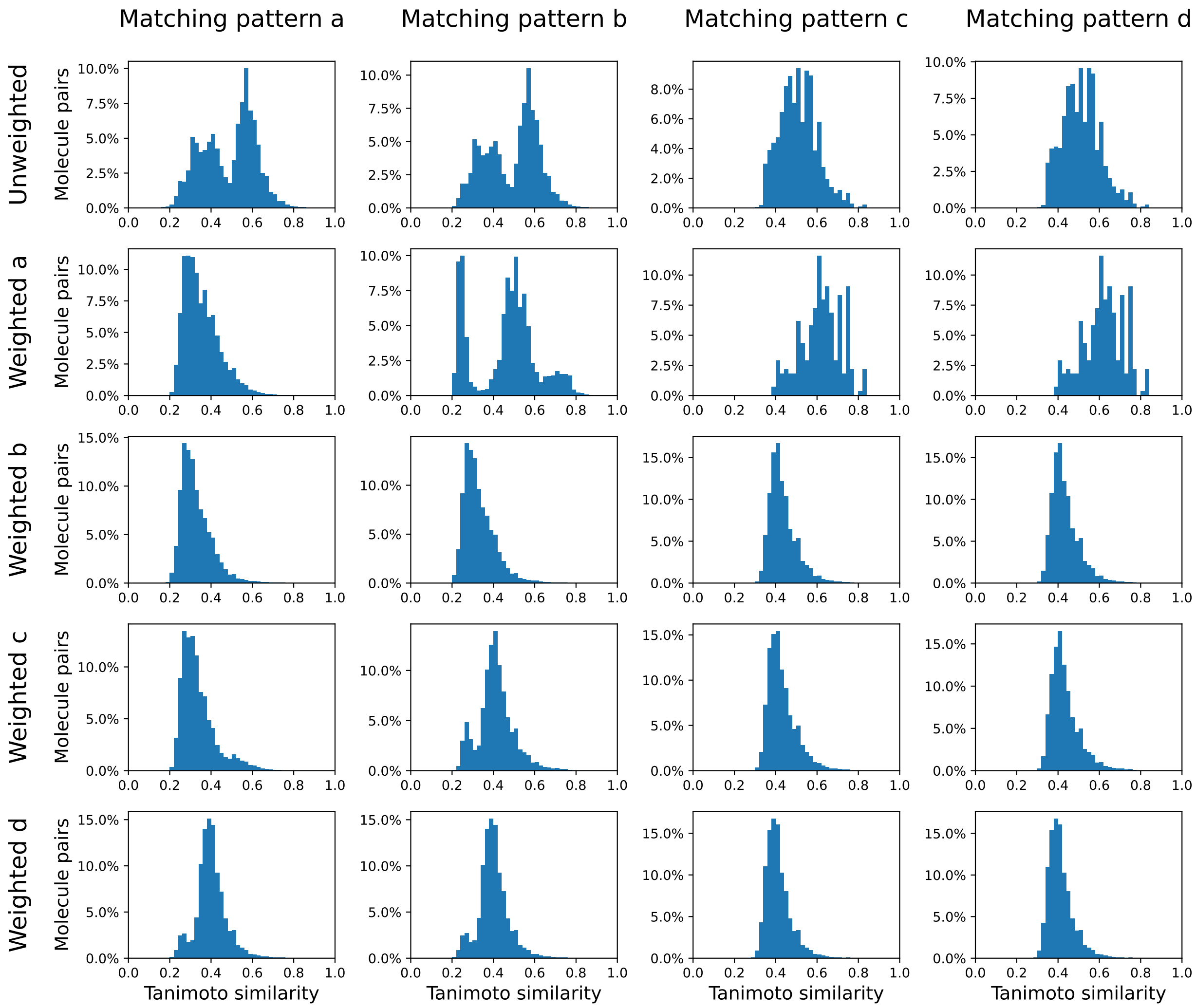

Figure S6: The pairwise Tanimoto similarity values[7] of all result molecules that contain the respective patterns *a*-*d* from the different G43 analog searches in the REAL Space[2]. The columns show which pattern the molecules match, and the rows show which pattern was used for weighting during the search. The similarity values were calculated based on the ECFP_4[4] fingerprint.

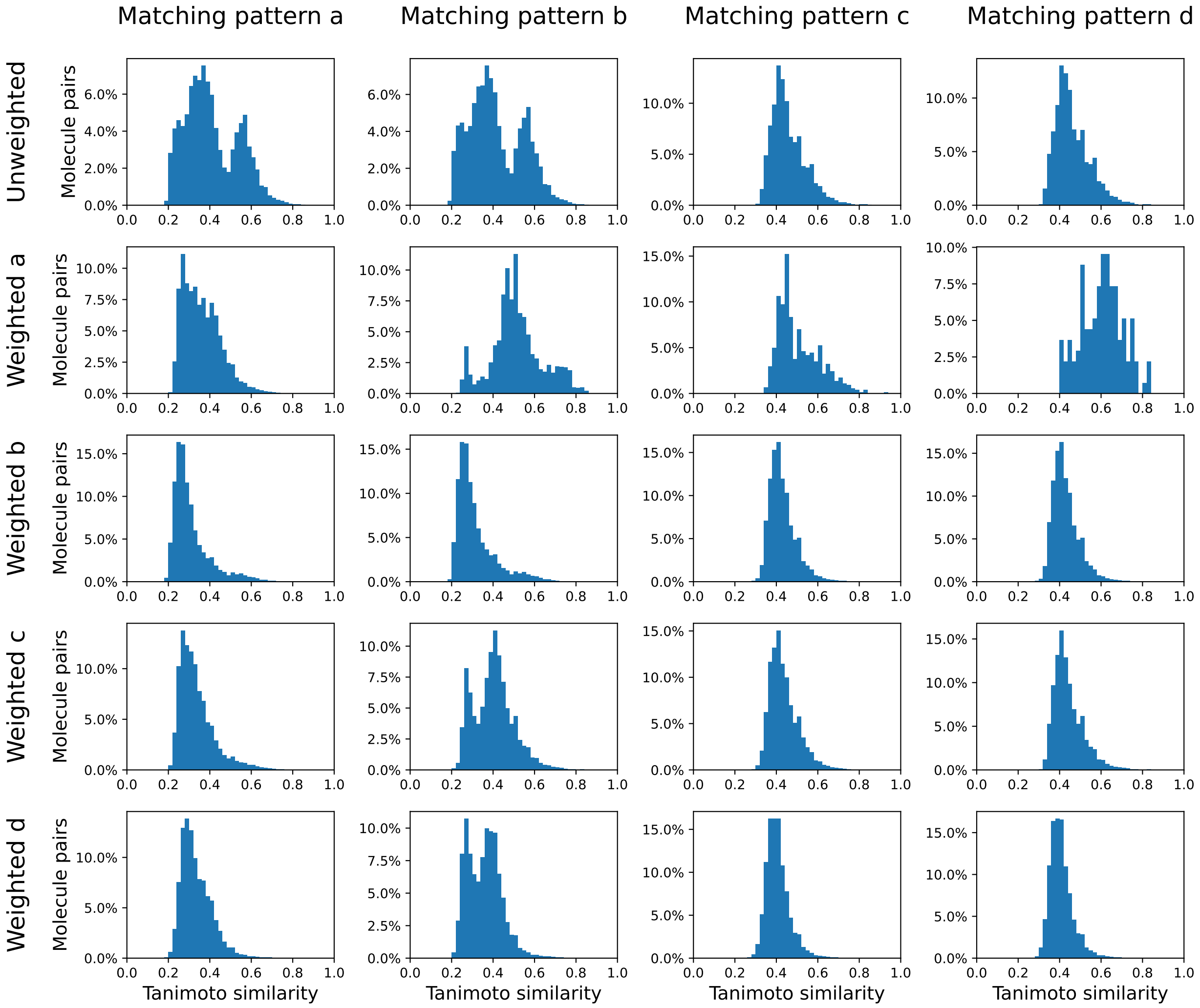

Figure S7: The pairwise Tanimoto similarity values[7] of all result molecules that contain the respective patterns *a*-*d* from the different G43 analog searches in the SAVI Space[5]. The columns show which pattern the molecules match, and the rows show which pattern was used for weighting during the search. The similarity values were calculated based on the ECFP_4[4] fingerprint.

References

(1) Zdrazil, B. et al. The ChEMBL Database in 2023: a drug discovery platform spanning multiple bioactivity data types and time periods. *Nucleic Acids Research* **2023**, *52*, D1180–D1192.

(2) Enamine REAL Space. https://enamine.net/compound-collections/real-compounds/real-space-navigator, accessed 2025-09-29.

(3) Bellmann, L.; Penner, P.; Rarey, M. Connected Subgraph Fingerprints: Representing Molecules Using Exhaustive Subgraph Enumeration. *Journal of Chemical Information and Modeling* **2019**, *59*, 4625–4635.

(4) Rogers, D.; Hahn, M. Extended-Connectivity Fingerprints. *Journal of Chemical Information and Modeling* **2010**, *50*, 742–754.

(5) Korn, M.; Judson, P.; Klein, R.; Lemmen, C.; Nicklaus, M. C.; Rarey, M. SAVI Space—combinatorial encoding of the billion-size synthetically accessible virtual inventory. *Scientific Data* **2025**, *12*, 1064.

(6) Bemis, G. W.; Murcko, M. A. The Properties of Known Drugs. 1. Molecular Frameworks. *Journal of Medicinal Chemistry* **1996**, *39*, 2887–2893.

(7) Jaccard, P. Lois de distribution florale dans la zone alpine. *Bulletin de la Société vaudoise des sciences naturelles* **1902**, *38*, 69–130.
