# Supplementary figures and images for: Guiding Similarity Search in Chemical Fragment Spaces with Weighted Fingerprints
Source: J Chem Inf Model. 2026 Feb 10;66(4):2220–9. doi: 10.1021/acs.jcim.5c02952 (PMC12933719; doi:10.1021/acs.jcim.5c02952)

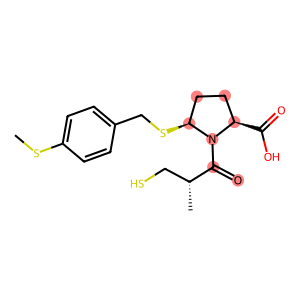

Supplement: Supplementary file 2 [file ci5c02952_si_002.zip › Supporting_Information_Data/Statistical_Validation/Example_Results/SAVI_Space_CHEMBL525967/marked_atoms1.png]

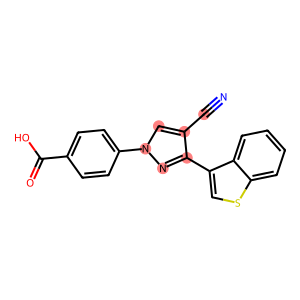

Supplement: Supplementary file 2 [file ci5c02952_si_002.zip › Supporting_Information_Data/Statistical_Validation/Example_Results/REAL_Space_CHEMBL3663141/marked_atoms1.png]
